# Genome-wide analysis of plant nat-siRNAs reveals insights into their distribution, biogenesis and function

**DOI:** 10.1186/gb-2012-13-3-r20

**Published:** 2012-03-22

**Authors:** Xiaoming Zhang, Jing Xia, Yifan E Lii, Blanca E Barrera-Figueroa, Xuefeng Zhou, Shang Gao, Lu Lu, Dongdong Niu, Zheng Chen, Christy Leung, Timothy Wong, Huiming Zhang, Jianhua Guo, Yi Li, Renyi Liu, Wanqi Liang, Jian-Kang Zhu, Weixiong Zhang, Hailing Jin

**Affiliations:** 1 Department of Plant Pathology and Microbiology, Center for Plant Cell Biology and Institute for Integrative Genome Biology, University of California, Riverside, CA 92521, USA; 2 Department of Computer Science and Engineering, Washington University in St Louis, St Louis, MO 63130, USA; 3 Department of Botany and Plant Sciences, Center for Plant Cell Biology and Institute for Integrative Genome Biology, University of California, Riverside, CA 92521, USA; 4 Instituto de Biotecnologia, Universidad del Papaloapan, Tuxtepec Oaxaca 68301, Mexico; 5 Peking-Yale Joint Center for Plant Molecular Genetics and Agrobiotechnology, The State Key Laboratory of Protein and Plant Gene Research, College of Life Sciences, Peking University, Beijing, 100871, China; 6 Department of Plant Protection, Nanjing Agriculture University, Nanjing, 210095, China; 7 Department of Horticulture and Landscape Architecture, Purdue University, West Lafayette, IN 47907, USA; 8 School of Life Sciences and Biotechnology, Shanghai Jiao Tong University, Shanghai, 200240, China; 9 Department of Genetics, Washington University School of Medicine, St Louis, MO 63110, USA

## Abstract

**Background:**

Many eukaryotic genomes encode *cis*-natural antisense transcripts (*cis*-NATs). Sense and antisense transcripts may form double-stranded RNAs that are processed by the RNA interference machinery into small interfering RNAs (siRNAs). A few so-called nat-siRNAs have been reported in plants, mammals, *Drosophila*, and yeasts. However, many questions remain regarding the features and biogenesis of nat-siRNAs.

**Results:**

Through deep sequencing, we identified more than 17,000 unique siRNAs corresponding to *cis*-NATs from biotic and abiotic stress-challenged *Arabidopsis thaliana *and 56,000 from abiotic stress-treated rice. These siRNAs were enriched in the overlapping regions of NATs and exhibited either site-specific or distributed patterns, often with strand bias. Out of 1,439 and 767 *cis*-NAT pairs identified in *Arabidopsis *and rice, respectively, 84 and 119 could generate at least 10 siRNAs per million reads from the overlapping regions. Among them, 16 *cis*-NAT pairs from *Arabidopsis *and 34 from rice gave rise to nat-siRNAs exclusively in the overlap regions. Genetic analysis showed that the overlapping double-stranded RNAs could be processed by Dicer-like 1 (DCL1) and/or DCL3. The DCL3-dependent nat-siRNAs were also dependent on RNA-dependent RNA polymerase 2 (RDR2) and plant-specific RNA polymerase IV (PolIV), whereas only a fraction of DCL1-dependent nat-siRNAs was RDR- and PolIV-dependent. Furthermore, the levels of some nat-siRNAs were regulated by specific biotic or abiotic stress conditions in *Arabidopsis *and rice.

**Conclusions:**

Our results suggest that nat-siRNAs display distinct distribution patterns and are generated by DCL1 and/or DCL3. Our analysis further supported the existence of nat-siRNAs in plants and advanced our understanding of their characteristics.

## Background

Gene regulation through small non-coding RNAs has been recognized as an important mechanism for almost all cellular processes in eukaryotes. Most small RNAs (sRNAs) are processed by RNase III-type ribonuclease Dicer-like (DCL) proteins, and incorporated into Argonaute (AGO) proteins to guide gene silencing at the transcriptional level through DNA or histone modification, and at the posttranscriptional level by mRNA cleavage and degradation or translational repression [1-5]. Based on their precursor structures and biogenesis, sRNAs can be categorized into microRNAs (miRNAs) and small-interfering RNAs (siRNAs). miRNAs are derived from hairpin-structured precursors and do not depend on RNA-dependent RNA polymerases (RDRs) or RNA amplification mechanisms, whereas endogenous siRNAs (endo-siRNA) are generated from long double-stranded RNAs (dsRNAs) as a result of antisense transcription or the activity of plant-specific RNA polymerases such as RNA polymerase (Pol)IV and RDRs [6-8].

Different classes of endogenous siRNAs have been described in *Arabidopsis *based on their distinct characteristics and biogenesis pathways [3,9]. The 24-nucleotide heterochromatic siRNAs (hc-siRNAs) or repeat-associated siRNAs (ra-siRNAs) are generated by the PolIV/RDR2/DCL3 pathway and function in RNA-directed DNA methylation and histone modifications [2,10-12]. Trans-acting siRNAs (ta-siRNAs) are 21 nucleotides in length and require DCL4, RDR6, and miRNA-mediated cleavage that triggers the phased processing [3]. Long siRNAs (lsiRNAs) are 30 to 40 nucleotides in length and are dependent on DCL1, HEN1, RDR6 and PolIV [13]. lsiRNAs most likely induce decapping and 5' to 3' mRNA degradation of their targets [13]. Several *cis*-natural antisense transcripts (*cis*-NATs) have been reported to also generate siRNAs [14-18]. These so-called nat-siRNAs are induced by abiotic and biotic stresses [16,17,19] or accumulate in specific developmental stages [14,15]. The biogenesis of salt- and bacterium-induced nat-siRNAs appeared to require DCL1 and/or DCL2, RDR6, and PolIV in *Arabidopsis *[16,17]. Moreover, the expression of *ARIADNE14 *is de-repressed in *dcl1*, *hen1*, *hyl1*, *sde4*, *rdr2 *and *sgs3*, suggesting that the nat-siRNAs generated from the *ARIADNE14*/*KOKOPELLI *overlapping pair is dependent on DCL1, HEN1, HYL1, RDR2, SGS3 and PolIV [14]. The overlapping regions of some *cis*-NATs in mouse and *Drosophila *also produce endo-siRNAs, which are processed by Dcr-2 and loaded into AGO2 [20-24]. nat-siRNAs have also been found in budding yeast and *Schistosoma japonicum *[25,26]. Despite these reports, there remains a need for conclusive evidence for the occurrence of nat-siRNAs in plants [27], because their features are not well understood and a general model for their generation is still not available.

Here we report the identification of 33,826 and 162,397 siRNAs corresponding to 17,141 and 56,209 unique sequencing reads that were derived from *cis*-NATs of biotic and abiotic stress-challenged *Arabidopsis *and abiotic stress-treated rice, respectively. These siRNAs, displaying either a distributed or a site-specific pattern, were enriched in the overlapping regions and often exhibited strand bias. Genetic analysis revealed that nat-siRNAs in *Arabidopsis *were mainly generated by DCL1 and/or DCL3, and a subgroup of them was RDR- and PolIV-dependent. Furthermore, accumulation of some nat-siRNAs was regulated by biotic and abiotic stress conditions, which may contribute to plant responses to environmental stresses.

## Results

### Natural antisense transcripts and *cis*-NAT-derived siRNAs in *Arabidopsis *and rice

We sequenced a total of 21 sRNA libraries prepared from biotic and abiotic stress-treated *Arabidopsis *and abiotic stress-challenged *Oryza sativa *plants. Biotic stress datasets for *Arabidopsis *included infection of non-pathogenic *Pseudomonas syringae pv tomato *(*Pst*) DC3000 *hrcC*^-^, a virulent strain *Pst *DC3000 carrying an empty vector, and an avirulent strain *Pst *DC3000 carrying an effector gene, *avrRpt2*, as previously described [28-30]. Abiotic stresses on *Arabidopsis *and rice included salt, cold and drought treatments (see Materials and methods). We obtained a total of more than 24.6, 23.2 and 30.9 million raw sequencing reads from these biotic and abiotic stress-treated *Arabidopsis *and abiotic stress-treated rice small-RNA libraries, respectively (Additional file 1). After trimming adaptor sequences and discarding reads shorter than 17 nucleotides or longer than 28 nucleotides or of low quality, we obtained 13,985,938, 14,664,923 and 17,446,592 reads from the biotic and abiotic stress-treated *Arabidopsis *libraries and the abiotic stress-treated rice libraries that perfectly match to the *Arabidopsis *and rice genomes or cDNAs, respectively (Additional file 1). Among all the small-RNA species profiled, 24-nucleotide sRNAs were the most abundant size class (Additional file 2a). The 5'-first nucleotides of sRNAs preferentially are adenosine or uracil (Additional file 2c). In addition to a large number of miRNAs [28,29], we discovered many new endogenous siRNAs belonging to different siRNA categories. In this study, we focused on siRNAs that correspond to *cis*-NATs.

Using the *Arabidopsis *genome annotation (TAIR/NCBI version 8.0) and the rice genome annotation (MSU RGAP 6.1), we searched for pairs of genes in *Arabidopsis *and rice that overlapped more than 25 nucleotides at the same genomic loci. We thus identified 1,439 and 767 pairs of *cis*-NATs in *Arabidopsis *and rice, respectively (Table 1). *cis*-NATs can be further categorized into three groups: convergent (3'-3' overlap), divergent (5'-5' overlap), and enclosed, in which one transcript is entirely encompassed by the other. Most *cis*-NATs (1,126 and 560 pairs in *Arabidopsis *and rice, respectively) are arranged in convergent orientation (Table 1). We excluded 65 and 10 *cis*-NAT pairs in which at least one gene encodes rRNA, tRNA, small nuclear RNA (snRNA), small nucleolar RNA (snoRNA), miRNA, ta-siRNA, or transposons in *Arabidopsis *and rice, respectively, from subsequent analyses. These RNA genes or transposons are considered 'hot spots' of RNA degradation or small RNA generation and may mask the real distribution of nat-siRNAs. We also analyzed the NAT genes targeted by miRNAs to eliminate the possibility that the sRNAs that matched to the transcripts are secondary siRNAs triggered by 22-nucleotide miRNAs [31,32]. We found that At2g33810 in the At2g33810/At2g33815 pair was a target of miR156 and At1g53230 in the At1g53230/At1g53233 pair was a target of miR319. However, neither miR156 nor miR319 is 22 nucleotides, and the siRNAs generated from At2g33810 and At1g53230 were not arranged in a phasing pattern, which is a characteristic pattern of miRNA-triggered secondary siRNAs. Therefore, we retained At2g33810/At2g33815 and At1g53230/At1g53233 in our list. A total of 1,374 NAT pairs in *Arabidopsis *were used in this study. Among them, 186 genes (6.8% of 2,748 NAT genes) are annotated as 'other RNAs'. They are most likely non-coding RNAs or RNAs with potential to encode very short proteins or peptides.

**Table 1:** Different types of *cis*-NATs identified in *Arabidopsis *and rice and *cis*-NATs that generated *cis*-nat-siRNAs

Type of *cis*-NAT pairs	All pairs	All pairs analyzed	Pairs with >10 siRNA reads in OR (%)^a^	Pairs with siRNAs only in the OR (%)^b^	Pairs with siRNAs >2-fold enriched in OR (%)^b^	Pairs with siRNAs of strand bias (>2-fold) (%)^b^
*Arabidopsis*						
Convergent	1,126	1,103	23 (2.1%)	5 (21.7%)	9 (37.5%)	13 (54.2%)
Divergent	142	127	22 (17.3%)	4 (18.2%)	5 (20.0%)	11 (44.0%)
Enclosed	171	144	39 (27.1%)	7 (17.9%)	10 (23.8%)	22 (52.4%)
Total	**1,439**	**1,374**	**84 (6.1%)**	**16 (19.0%)**	**24 (26.4%)**	**46 (50.5%)**
						
Rice						
Convergent	560	551	68 (12.3%)	20 (29.4%)	31 (45.6%)	36 (52.9%)
Divergent	100	99	12 (12.1%)	1(8.3%)	3 (25.0%)	4 (33.3%)
Enclosed	107	107	39 (36.4%)	13 (33.3%)	11 (28.2%)	23 (59.0%)
Total	**767**	**757**	**119 (15.7%)**	**34 (28.6%)**	**45 (37.8%)**	**63 (52.9%)**

OR, overlap region of a *cis*-NAT transcript pair. ^a ^The percentage of *cis*-NAT pairs in all *cis*-NAT pairs analyzed. ^b ^The percentage of the specified *cis*-NAT pairs in the corresponding regions with siRNAs.

We searched our small RNA deep-sequencing libraries for reads that matched perfectly (that is, no mismatches) to the *cis*-NATs. We found that 84 (6.1% of 1,374) and 119 (15.7% of 757) of the *Arabidopsis *and rice *cis*-NAT pairs, respectively, gave rise to at least 10 sRNAs per one million total genome-mapped reads from the overlapping regions of the *cis*-NATs in all the libraries (Table 1; Additional file 3). Interestingly, among the 84 *Arabidopsis *NAT pairs, 54 pairs have one transcript (32.1% of 168 genes) annotated as 'other RNAs' (Additional file 4), suggesting that non-coding antisense RNAs are more likely to generate siRNAs. A total of 33,826 and 162,397 siRNAs corresponding to 17,059 and 56,209 unique reads were derived from *cis*-NATs in *Arabidopsis *and rice, respectively (Table 2). As shown in Additional file 2, nat-siRNAs are mainly 21 and 24 nucleotides in length, whereas in the total small RNA population, the 24-nucleotide siRNAs are the most abundant species. The first-nucleotide distribution for the nat-siRNAs was similar to that of the total sRNAs (Additional file 2).

**Table 2:** The numbers of nat-siRNAs from the entire regions of *cis*-NATs, overlap regions of *cis*-NATs, introns in entire regions of *cis*-NATs and introns in overlap regions of *cis*-NATs in *Arabidopsis *and rice

	*Arabidopsis*		Rice	
		
Origin of nat-siRNAs	Total	Unique	Total	Unique
Entire NATs	33,826	17,059	162,397	56,209
Introns in entire NATs	14,500	6,988	88,605	29,654
OR of NATs	10,429	6,532	27,852	10,761
Introns in OR of NATs	4,367	2,350	14,207	5,494

Raw reads of siRNAs that perfectly mapped to the *Arabidopsis *and rice genomes were analyzed here. OR, overlap region.

**Figure 1:**
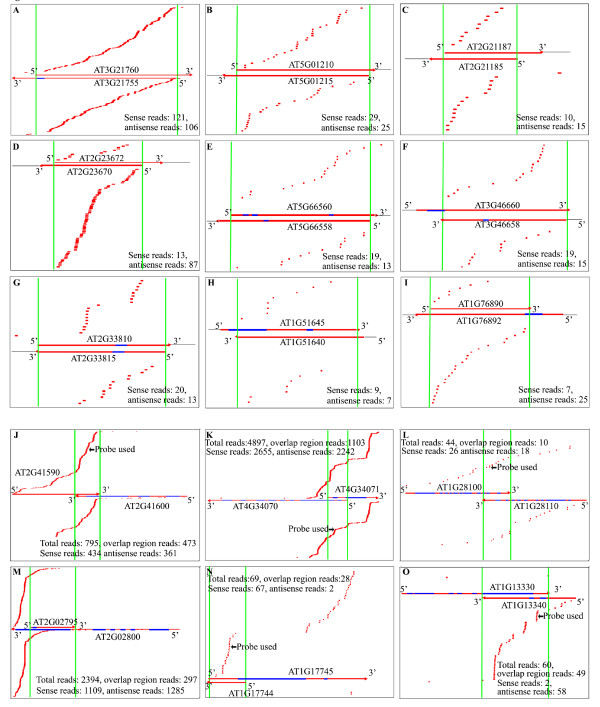
**Distinct distribution patterns of *Arabidopsis *nat-siRNAs**. **(a-i) ***Arabidopsis *nat-siRNAs displaying a distributed pattern and exclusively derived from the overlapping regions. **(j-o) ***Arabidopsis *nat-siRNAs displaying a distributed pattern but derived from both the overlap region and non-overlap region. sRNAs matching the upper and lower strands are displayed above and below the NAT pairs, respectively. The red regions on the gene model represent exons, whereas the blue regions represent introns. The region between the green lines represents the overlapping region of the NATs. siRNAs probed are indicated by black arrows.

**Figure 2:**
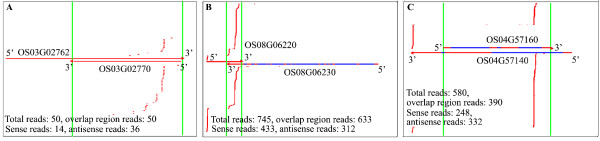
**Distribution of rice nat-siRNAs with distribution pattern OS03G02762/OS03G02770 (a) and OS08G06220/OS08G06230 (b) or site-specific pattern OS04G57140/OS04G57160 (c)**. Strands above or under the NAT pairs represent sRNAs positively or negatively mapping to the upper strands. In the gene model, exons and introns are indicated by red regions and blue regions, respectively. The overlapping regions of the NATs are indicated by green lines.

Within the three types of *cis*-NATs, enclosed *cis*-NATs have the highest percentages for producing siRNAs, that is, 39 (27.1% of 144) and 39 (36.4% of 107) of the enclosed *cis*-NATs in *Arabidopsis *and rice, respectively, generated siRNAs (Table 1). Among the 84 and 119 *cis*-NAT pairs that produce more than 10 siRNAs per million reads in the overlapping regions in *Arabidopsis *and rice, respectively, 16 and 34 pairs (19.0% and 28.6%) have siRNAs exclusively in the overlapping regions (Table 1, Figures 1a-i and 2a). To determine if siRNAs were truly enriched in the overlapping regions of NATs, we took into consideration the length differences between the overlapping and non-overlapping regions of *cis*-NATs by comparing the densities of siRNAs in these two regions. A statistical test showed that the density of siRNAs in the overlapping regions was more than six times that in the non-overlapping regions, with *P*-values of 0.0077 and 0.0242 in *Arabidopsis *and rice, respectively (see Materials and methods). To further explore whether *cis*-NATs have a higher likelihood to give rise to siRNAs than other protein-coding genes, we computed the densities of sRNAs in the 3'-UTR overlapping regions of convergent *cis*-NATs and 5'-UTR overlapping regions of divergent *cis*-NATs, and the siRNA densities in the 3'-UTRs and 5'-UTRs of protein-coding genes that were not involved in NATs, respectively (see Materials and methods). We then compared the density of siRNAs in the convergent *cis*-NATs with the density of siRNAs in the 3'-UTR of non-NAT genes in *Arabidopsis*; similarly, we compared divergent *cis*-NATs with the 5'-UTR of non-NAT genes. The densities of siRNAs in divergent 5' and convergent 3' *cis*-NATs were statistically greater than that of the 5'- and 3'-UTRs of non-NAT genes, with *P*-values of 0.0366 and 0.0438, respectively. These results confirmed that *cis*-NATs were more likely than non-NAT genes to generate siRNAs. This is further supported by the study of Argonaute-associated sRNAs, which indicated that *cis*-NATs generated 2.29 times more sRNAs than other gene transcripts [33]. Our results were also consistent with that of animal endo-siRNAs, which are also enriched in the overlapping regions of *cis*-NATs, for example, more than ten-fold enrichment in *Drosophila *[21-23,25,34].

**Figure 3:**
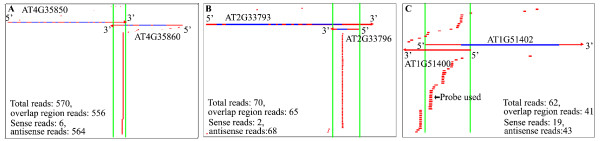
**Site-specific patterns of *Arabidopsis *nat-siRNAs At4g35850/At4g35860 (a), At2g33793/At2g33796 (b), and At1g51400/At1g51402 (c)**. siRNAs that positively or negatively map to the upper genes are present above or under the gene pair, respectively. The introns of *cis*-NATs are represented by blue strands and overlapping regions of *cis*-NATs are shown by green lines. The black arrow represents the siRNAs detected by Northern blot.

The distribution of siRNAs along the *cis*-NATs display two distinct patterns, one is the distributed pattern with siRNAs scattered along the overlapping regions or across the entire transcripts (Figures 1 and 2a, b), and the other is the site-specific pattern with siRNAs derived from one or a few specific sites (Figures 2c and 3) (see Materials and methods for additional details). We identified 9 site-specific patterns from *Arabidopsis *and 23 from rice (Additional files 5 and 6). Because RNA secondary structure analysis at these specific sites using RNAFold [35] revealed no obvious stem-loop structures, these regions were unlikely to harbor new miRNA genes or nat-miRNA genes as described [36], although we are aware of the limitation of RNAfold. Moreover, 46 and 63 (50.5% and 52.9%) *cis*-NAT pairs in *Arabidopsis *and rice, respectively, exhibited strand bias in generating nat-siRNAs with different directions (with more than two-fold change) (Table 1**)**.

### Requirement of DCL1 and/or DCL3 for the accumulations of nat-siRNAs

**Figure 4:**
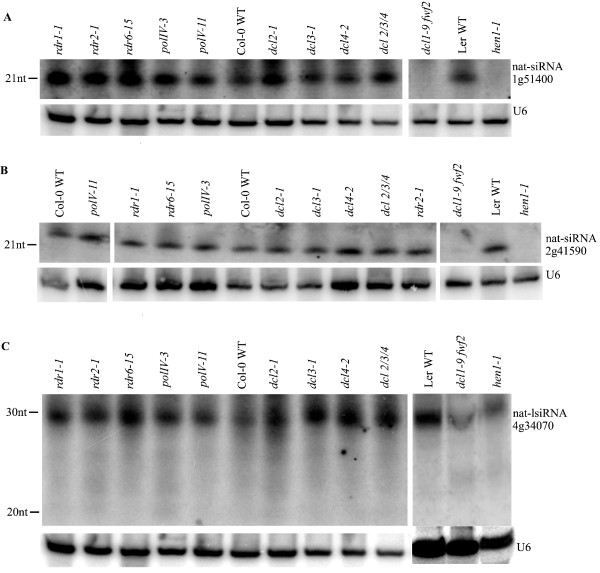
**Biogenesis of nat-siRNAs that depend on DCL1**. **(a-c) **Accumulation of nat-siRNA1g51400 (a), nat-siRNA2g41590 (b) and nat-lsiRNA4g34070 (c) is shown in various *dcl*, *rdr*, *polIV *and *polV *mutants. Total RNA (75 to 100 μg) was used for Northern blot analysis. LNA probes complementary to each nat-siRNA were used. U6 was an internal control to show equal loading. The same results were obtained from two biological replicates.

To experimentally characterize the nat-siRNAs, we examined the expression of 15 new nat-siRNAs identified in *Arabidopsis *with relatively high numbers of reads. In addition to nat-siRNAATGB2, another 6 out of the 15 nat-siRNAs that we examined showed clear siRNA signals by small RNA Northern blot analysis using locked nucleic acid (LNA) probes (indicated by black arrows in Figures 1 and 3; probes used are listed in Additional file 7), which enabled us to study their biogenesis by examining their expression in small RNA pathway mutants. The biogenesis of the site-specific nat-siRNA generated from the divergent pair At1g51400/At1g51402 (referred to as nat-siRNA1g51400; Figure 3c) was dependent on DCL1 and HEN1, but did not require any RDRs or PolIV (Figure 4a). Similarly, the nat-siRNA generated from the convergent pair At2g41590/At2g41600 with a distributed pattern (referred to as nat-siRNA2g41590; Figure 1j) was also DCL1-dependent but RDR- and PolIV-independent (Figure 4b). Both nat-siRNAs were 21 nucleotides in length. Previous studies have shown that the biogenesis of bacterial-induced nat-siRNAATGB2, and the accumulation of some of the salt-induced nat-siRNASRO5 and development-associated nat-siRNAKPL also depended on DCL1 [14,16,17].

**Figure 5:**
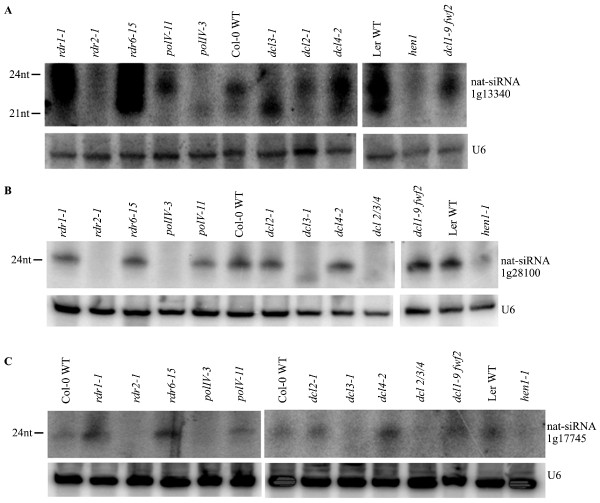
**Biogenesis of nat-siRNAs that depend on DCL3**. **(a-c) **Accumulation of nat-siRNA1g13340 (a), nat-siRNA1g28100 (b) and nat-siRNA1g17745 (c)Total RNA (75 to 100 μg) of distinct *dcl*, *rdr*, *polIV *and *polV *mutants and corresponding WT are probed by LNA probes indicated in Figures 1 and 3. U6 was used as a loading control. Similar results were obtained in two biological repeats.

We found that the convergent NAT pair At1g13330/At1g13340 (Figure 1o) could produce two classes of siRNAs, 21-nucleotide and 24-nucleotide siRNAs (referred to as nat-siRNA1g13340) from the same site (Figure 5a). Genetic analysis revealed that the 21-nucleotide siRNAs were DCL1-dependent, whereas the 24-nucleotide siRNAs were DCL3-dependent. Furthermore, we found that two other 24-nucleotide nat-siRNAs with a distributed pattern, nat-siRNA1g28100 generated from NAT pair At1g28100/At1g28110 (Figure 1l) and nat-siRNA1g17745 generated from NAT pair At1g17744/At1g17745 (Figure 1n), were also dependent on DCL3 (Figure 5b, c). Interestingly, all the DCL3-dependent 24-nucleotide nat-siRNAs examined are dependent on RDR2 and PolIV (Figure 5), while only some of the 21-nucleotide nat-siRNAs are dependent on RDRs and PolIV [14,16,17]. Deep sequencing results showed that 82 out of the 84 *cis*-NAT pairs gave rise to two classes of siRNAs, 20- to 22-nucleotide and 23- to 28-nucleotide siRNAs in the overlapping regions, while the remaining two pairs gave rise to the 20- to 22-nucleotide class only (Additional file 4). Thus, our results suggested that the *cis*-NATs have the potential to be processed by both DCL1 and DCL3.

### nat-siRNAs generated from introns

In both plants and animals, nat-siRNAs are derived from NAT mRNAs and mainly function post-transcriptionally [16,17,23]. This was also supported by our observation that some NATs with distributed nat-siRNAs predominantly appeared in exonic regions of protein coding genes, for example, At2g33810/At2g33815, At1g13330/At1g13340, OS08g06220/OS08g06230, and At4g35850/At4g35860 (Figures 1g, o, 2b and Figure 3a). However, based on the *Arabidopsis *genome annotation (TAIR version 8), we found that 383 of the 1,374 *cis*-NAT pairs (27.9%) contain introns in the overlapping regions. Among them, 45 (11.7%) have siRNAs mapped to introns. Examples are shown for At2g41590/At2g41600, AT434070/At4g34071 and At2g02795/At2g02800 (Figure 1j, k, m). In rice, 346 of 757 (45.7%) *cis*-NAT pairs contain introns in the overlapping regions; one example is shown for OS04g57140/OS04g57160 in Figure 2c. A substantial number of nat-siRNAs originated from such introns within NATs, as listed in Table 2 and Additional files 8 and 9. Similarly, it has been reported that a large number of siRNAs are also generated from the introns of several *cis*-NATs in *Drosophila *[23]. It is possible that the intronic nat-siRNAs are generated from pre-mRNAs in the nucleus before the introns are spliced out.

To confirm these intron-derived nat-siRNAs, we validated the candidates in small RNA biogenesis mutants. Although the total number of siRNAs from the entire overlapping regions might be large, the reads for individual siRNAs were still low. Most of the siRNAs examined were below the detection level of Northern blot analysis except one siRNA that is derived from an intron-exon junction of At4g34070 in the overlapping region of the divergent NAT pair At4g34070/At4g34071 (Figure 1k). We detected a distinct 30-nucleotide siRNA band using a 24-nucleotide probe. Because we used a 18- to 26-nucleotide size-fractionated small-RNA population for constructing our small-RNA libraries, lsiRNAs were excluded from our small-RNA sequencing dataset. To determine whether it was a new lsiRNA like the ones we detected before [13], or just a degradation product, we examined the dependence of this 30-nucleotide siRNA on components of the small RNA biogenesis pathways. We found that the generation of this siRNA depended on DCL1 and HEN1, but not on DCL3, or any RDRs, PolIV or PolV, indicating that it was a new lsiRNA but not a hc-siRNA (Figure 4c). It is worth noting that the reported AtlsiRNA-1 is also dependent on DCL1 and HEN1 [13]. These results suggested that a subgroup of nat-siRNAs was likely to be generated from pre-mRNAs.

### Some nat-siRNAs regulate the expression of their cognate NAT mRNAs in cis

We found that siRNAs from some *cis*-NATs accumulated to different levels in different libraries (Additional files 10 and 11). Eighty *cis*-NAT pairs in *Arabidopsis *and 37 *cis*-NAT pairs in rice displayed a more than two-fold difference of siRNA accumulation in the overlap regions under at least one stress condition as compared with mock-treated samples. This result suggested that these nat-siRNAs were likely to regulate expression of the NATs under stress conditions, although more experiments are needed to validate and confirm these changes.

To determine the regulatory function of nat-siRNAs on their NAT transcripts in *cis*, we performed computational analysis using *dcl1*-*7 *and *dcl3*-*1 *(inflorescence tissue) microarray data generated by the Carrington lab [37] (see Materials and methods), because DCL1 and DCL3 are responsible for nat-siRNA biogenesis. Of the 84 pairs of *cis*-NAT genes, 93 genes from 78 pairs were present on the 22-K microarray chip. Of the 93 *cis*-NAT genes, 22 (23.7%) exhibited a greater than 1.5-fold up-regulation in the *dcl1*-*7 *mutant compared to the wild type (WT) control (*P*-value < 0.05; Additional file 12). As a comparison, only 176 out of 2,215 (7.9%) total NAT genes were up-regulated. Although we cannot absolutely rule out the possible small RNA-independent function of DCL1 on the accumulation of miRNA targets [38], the result that more siRNA-associated NATs (23.5%) were regulated by DCL1 than total NATs (7.9%) strongly suggests that these small RNA-producing *cis*-NATs were more prone to down-regulation by DCL1-dependent nat-siRNAs. However, we did not find any of the 93 NAT genes that were up-regulated more than 1.5-fold in *dcl3*, suggesting that the DCL3-dependent nat-siRNAs may not be directly involved in the expression regulation of the NAT transcripts.

**Figure 6:**
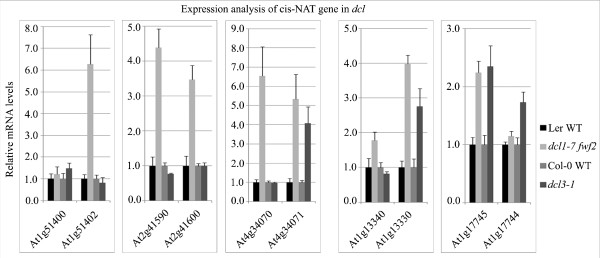
**Expression of the NAT transcripts is increased in *dcl1*-*7 fwf2 *and *dcl3*-*1 *mutants**. Expression was examined by quantitative RT-PCR and *Actin2 *was used as an internal control. Total RNA (5 μg) was treated with DNaseI and then subjected to reverse transcription. Error bars indicate standard deviations derived from three technical replicates. Similar results were obtained from two biological replicates.

To confirm the results from the microarray analysis and to examine the expression of some siRNA-targeting NAT transcripts that were not present on the chip or were not identified from the analysis, we used real-time RT-PCR to examine their expression in the WT, *dcl3*-*1 *and *dcl1*-*7 fwf2 *double mutant plants. We chose to use the double mutant instead of the *dcl1*-*7 *single mutant because the *fwf2 *mutant, which carries a mutation in the *ARF8 *gene [39,40], largely rescued the pleiotropy and fertility defects of *dcl1*-*7 *[41,42]. We found that the accumulation of At1g51402 and At1g13330 increased six and four times more, respectively, in the *dcl1*-*7 fwf2 *mutant than in WT plants (Figure 6), indicating that the antisense transcripts were no longer suppressed when nat-siRNA1g51400 and nat-siRNA1g13340 were not produced. For At2g41590/At2g41600 and At4g34070/At4g34071 pairs that produce siRNAs from both strands and have less strand bias, both sense and antisense transcripts were up-regulated in the *dcl1*-*7 fwf2 *mutant compared to the WT, suggesting that both transcripts were under the regulation of nat-siRNAs (Figure 6). Although it has been shown that the *arf8 *mutation does not interfere with miRNA-mediated gene silencing or RNAi or small RNA generation [39,40], it is still possible that up-regulation of these NAT transcripts in the *dcl1*-*7 fwf2 *double mutant is due to the *fwf2 *mutation that is sRNA-independent. To test this possibility, we also examined the expression levels of these NAT genes in the *fwf2 *single mutant. As shown in Additional file 13, in the *fwf2 *single mutant, there was no clear induction of these NAT transcripts except for At1g13340. Both transcripts of the At1g13340/Ag1g13330 pair were induced in the *dcl1*-*7*/*fwf2 *double mutant and At1g13340 was induced to a similar extent in both single and double mutants (about two-fold), suggesting that the up-regulation of At1g13340 was mainly due to the *arf8 *mutation whereas induction of At1g13330 was attributable to the de-repression of small RNA-mediated silencing by the mutation in DCL1. sRNAs generated from this pair had a strong strand bias (Figure 1o), were predominantly generated from At1g13340 and targeted At1g13330. These results correlated well with our real-time RT-PCR results. In summary, these data indicated that the expression of NAT transcripts was regulated by the small RNAs generated from DCL1.

We found that only three out of the ten transcripts that we tested showed an increase in the *dcl3*-*1 *mutant compared to WT (Figure 6), suggesting that some 24-nucleotide nat-siRNAs are still able to regulate the expression of NAT transcripts, although a majority of the NATs were not affected by the 24-nucleotide siRNAs.

To further analyze the function of these nat-siRNAs, we searched the publicly available deep sequencing datasets of the AGO-associated small-RNAs in *Arabidopsis *for the nat-siRNAs in the overlap regions we identified [43,44]. As shown in Additional file 14, the 20- to 22-nucleotide nat-siRNAs were mainly loaded into AGO2, the Argonaute protein that predominately binds 21-nucleotide sRNAs and contributes to anti-viral and anti-bacterial resistances [45-48]. Interestingly, the 23- to 26-nucleotide long nat-siRNAs were loaded into both AGO1 and AGO4, the Argonaute proteins responsible for mRNA cleavage and DNA methylation, respectively [49,50].

## Discussion

We identified 17,141 and 56,209 unique nat-siRNA sequences originating from *cis*-NATs in biotic and abiotic stressed *Arabidopsis *plants and abiotic stress-challenged rice, respectively. These siRNAs are enriched in the overlapping regions of *cis*-NAT pairs and display distributed or site-specific patterns.

Our biogenesis analysis suggested that the siRNAs from *cis*-NATs were dependent on DCL1 and/or DCL3. Deep sequencing results showed that 82 out of the 84 *cis*-NAT pairs that generated siRNAs in the overlapping regions gave rise to two classes of siRNAs, 20- to 22-nucleotide and 23- to 28-nucleotide nat-siRNAs, while only two pairs of NATs gave rise to one class of 20- to 22-nucleotide siRNAs (Additional file 4). The 20- to 22-nucleotide nat-siRNAs are generated by DCL1, whereas the 23- to 28-nucleotide nat-siRNAs were DCL3-dependent (Figures 4 and 5). We also provide an example that a DCL1-dependent 21-nucleotide nat-siRNA and a DCL3-dependent 24-nucleotide nat-siRNA were generated from the same site in the overlapping region of a NAT pair (Figure 5a). These two classes of sRNAs have also been observed in transgene silencing, viral RNA silencing and endogenous inverted repeat (IR) loci, where long double-stranded precursors or intermediates are formed [51-55]. Different kinds of transgenic constructs can generate different types of sRNAs: sense transgenes mainly generate 21- and 22-nucleotide siRNAs by DCL2 [56]; IR transgenes generate both 21- and 24-nucleotide siRNAs by DCL4 and DCL3, respectively, or mainly 21-nucleotide siRNAs by DCL4 [55-58]; DCL1 and DCL2 are also involved in the generation of siRNAs induced by IR transgenes [56]. Similarly, the 21-nucleotide, 22-nucleotide and 24-nucleotide endogenous siRNAs are processed by DCL4, DCL2 and DCL3, respectively, from endogenous IR loci [52,53]. DCL1 is required for the accumulation of 21- and 24-nucleotide siRNAs processed from IR transgenes or 22- and 24-nucleotide siRNAs processed from certain endogenous IR loci [53,59]. Viral RNAs are processed into 21- and 24-nucleotide siRNAs by DCL4 and DCL3, respectively, and DCL2 processes viral RNAs into 22-nucleotide siRNAs when DCL4 is suppressed or inactivated [60-64]. Furthermore, some endogenous miRNAs in *Arabidopsis *and rice also generate two distinct sizes of DCL1-dependent short miRNAs and DCL3-dependent long siRNAs or miRNAs at the same sites [28,65]. Our results suggest that, like the endogenous IR loci, transgene loci and viral replicons as well as *cis-*NAT sense and antisense transcripts form double-stranded RNAs that can potentially be processed by DCL1 and/or DCL3 to generate 20- to 22-nucleotide short siRNAs and 23- to 28-nucleotide long siRNAs, respectively. In this regard, the previously reported apparent DCL2 dependence of a 24-nucleotide nat-siRNA [17] is intriguing and needs to be re-examined.

By examining the expression of NAT transcripts that are complementary to the siRNAs and analyzing published microarray data, we found that many 21-nucleotide nat-siRNAs were able to regulate the accumulation of their target transcripts (Figure 6; Additional file 12), which was consistent with the function of reported 21-nucleotide nat-siRNAs processed by DCL1 [14,16,17]. However, only a small number of NATs targeted by siRNAs were up-regulated in the *dcl3 *mutant, suggesting that only a fraction of the 24-nucleotide class of siRNAs may regulate gene expression directly. Similar results were observed in the viral-derived 24-nucleotide siRNAs. Although 24-nucleotide viral siRNAs are as abundant as 21-nucleotide and/or 22-nucleotide siRNAs, they can neither silence viral RNAs nor suppress viral infection [60-64]. Endogenous 24-nucleotide siRNAs are mainly generated by the DCL3/RDR2/PolIV pathway, and are often associated with AGO4 and AGO6 and mainly direct DNA methylation or chromatin modification [49,50,54,66]. We found that the generation of the DCL3-dependent 23- to 28-nucleotide nat-siRNAs also required RDR2 and PolIV. Further studies are necessary to determine whether these 23- to 28-nucleotide nat-siRNAs can also direct DNA methylation or histone modification at their NAT loci.

We observed two major distribution patterns of nat-siRNAs - distributed and site-specific - in *Arabidopsis *and rice. We found that the site-specific nat-siRNAs tested were generated by DCL1. Because of its predominant role in processing hairpin structures into miRNAs, DCL1 is generally assumed to be capable of recognizing certain RNA secondary structures. The sense and antisense transcripts in a NAT pair are transcribed separately, and their overlapping regions may interact to form a dsRNA. In this partially dsRNA duplex, complex secondary structures may arise, which are recognized by the versatile DCL1 enzyme, and thus are processed to yield site-specific nat-siRNAs, or possibly distributed nat-siRNAs, depending on what secondary structures are formed and recognized by DCL1. It is also possible that these apparently site-specific nat-siRNAs may be generated across the whole overlapping region first as the distributed ones, but only specific siRNAs from particular sites are stable and thus accumulated, while the rest are degraded.

We showed that RDRs were not always required for nat-siRNA formation (Figure 4a-c), which suggested that the dsRNAs formed within the overlapping regions of the sense and antisense transcripts were sufficient for producing nat-siRNAs in such cases. As for the nat-siRNAs that do require RDRs [13,16,17], they were not completely eliminated in the *rdr *mutants. The partial dependence on RDRs suggested that these nat-siRNAs could be primarily generated from the dsRNA regions formed within overlapping sense-antisense transcripts, and then may be amplified by RDRs in a secondary amplification step. However, because the enrichment of *cis*-NAT-generated siRNAs compared to all genic siRNAs is relatively weak, one should not automatically assume that siRNA clusters corresponding to the *cis*-NAT genes are always caused by the *cis*-NAT configuration; it is possible that some siRNAs may coincidentally be in a *cis*-NAT region. Nevertheless, our observation of some siRNA clusters that appear exclusively in the overlapping regions of NATs strongly supports that at least some siRNAs are indeed caused by the *cis*-NAT configuration.

We also found that many nat-siRNAs were derived from introns or intron-exon junctions. It has been shown that the sense/antisense dsRNA pairs in vertebrates are processed in the nucleus but not the cytoplasm [67,68]. Therefore, some plant nat-siRNAs may also be produced in the nucleus before introns are spliced out from the NATs. Moreover, we found a new *Arabidopsis *lsiRNA, lsiRNA4g34070, which was generated from the exon-intron junction of a NAT pair. Similar to AtlsiRNA-1, lsiRNA4g34070 was processed by DCL1.

The extent of transcript overlap within each *cis*-NAT may differ from the TAIR annotations and requires careful examinations. For example, recent 3' rapid amplification of cDNA ends (RACE) experiments revealed that the major *SRO5 *transcripts (data not shown) are shorter than previously indicated [17], although the experiments also showed a longer *SRO5 *transcript that extends beyond the detected nat-siRNAs (GenBank accession JQ513374). Some genes such as *AGO3 *were annotated in the TAIR database as having no 3'-UTRs, but longer 3'-UTRs for some of these genes have been recovered by 3'-RACE in our labs and others. It appears that many genes have multiple forms of transcripts with various lengths of 3'-UTRs.

Plant nat-siRNAs have been shown to regulate gene expression in stress responses or under specific developmental stages [16,17]. In this study, we identified new nat-siRNAs that appeared to regulate the expression of their NAT mRNAs, which may contribute to gene expression reprogramming and fine-tuning in response to environmental stresses. NATs are widespread in eukaryotic organisms [19,69-72]. The expression of antisense transcripts may differ among different cell types or under different environmental conditions [19,73]. It is likely that different sets of nat-siRNAs may be generated from NATs in response to different developmental and environmental cues, and therefore play a broad regulatory role in gene expression under specific conditions. Several reported nat-siRNAs, such as nat-siRNASRO5, nat-siRNAATGB2 and nat-siRNAKPL, are induced under stress or appear at specific developmental stages and suppress the expression of their antisense transcripts post-transcriptionally [14,16-18,69]. Similar examples have also been observed in animal systems, for example, the siRNAs from the SIC34a1 gene pair are only generated from mouse kidney and testis [67]. Moreover, many *cis*-NAT gene pairs in mammalian brain, including the genes involved in Alzheimer's disease and LRRTM1/Ctnna2, a pair of *cis*-NAT genes that participates in schizophrenia, can generate nat-siRNAs [74]. A lot of these nat-siRNAs are up-regulated in olfactory discrimination training [74]. Therefore, most of the reported nat-siRNAs are generated under specific developmental or environmental conditions. In this study, we detected bacterial-induced nat-siRNAATGB2 [16], as well as another siRNA that is in phase with nat-siRNAATGB2 (Figure 3a; Additional file 10) by deep sequencing, whereas the developmental stage-specific nat-siRNA nat-siRNAKPL was not present in our database [14]. The salt-induced nat-siRNAs from the SRO5-P5CDH pair were also present in this dataset, but the number of reads was below our cutoff threshold (minimum ten reads per million total match reads in the overlapping region), so it was not included in our list. In order to be consistent with other abiotic stress treatment, the salt treatment condition in this study was different from that in the published work [17]. However, more SRO5-P5CDH siRNAs were detected from two other salt-treated small RNA libraries in a separate study (Additional file 15; Gene Expression Omnibus (GEO) accession number GSE33642). It is worth noting that our genome-wide analysis on nat-siRNA-targeting NAT transcripts used the microarray result generated from healthy inflorescences (GEO accession number GSE2473), which may miss some NAT transcripts that are regulated by siRNAs at a different developmental stage or under specific environmental conditions. In addition, we cannot rule out the possibility that some of the nat-siRNAs regulate the expression of their targets by translational inhibition and therefore cannot be directly detected at the mRNA level by microarray or real-time RT-PCR experiments.

## Conclusion

Our studies of plant sRNAs showed that those derived from plant *cis*-NATs were enriched in their overlap regions and *cis*-NATs are more likely to generate sRNAs than other transcripts. Taken together with previously published data on plants, *Drosophila *and yeast, our results strongly support the existence of nat-siRNAs in eukaryotic organisms. Our genetic analysis indicated that *cis*-NATs were processed by DCL1 and/or DCL3. By analyzing a large number of small RNA libraries from stress-challenged plants, we found that some nat-siRNAs were likely to respond to different environmental stresses, and therefore contributed to plant stress resistance, development or other cellular processes by regulating corresponding NAT mRNAs. nat-siRNA-mediated gene regulation is one of the regulatory mechanisms for at least a subgroup of *cis*-NATs.

## Materials and methods

### Plant materials

*Arabidopsis thaliana *mutants (*dcl1*-*9 fwf2*, *dcl1*-*7 fwf2*, *dcl2*-*1*, *dcl3*-*1*, *dcl4*-*2*, *hen1*, *rdr2*-*1*, *rdr1*-*1*, *rdr6*-*15*, *PolIV*-*3 *and *PolV*-*11*, *fwf2*) and their corresponding WT ecotypes were used in this study. Bacterial infection assays were performed on 4-week-old *Arabidopsis *grown at 23°C with 12 h light as described previously [28-30]. Abiotic stress assays were performed on 4-week-old *Arabidopsis *gl1 plants expressing Luciferase under the control of RD29 promoter grown at 23°C and 16 h light, and 3-month-old rice plants (*Oryza sativa cv. japonica*) grown at 28°C with 13 h light.

### Small-RNA library construction and deep sequencing

Bacteria infiltration was carried out on the leaves of 4-week-old *Arabidopsis *WT and mutant plants as described previously [46]. Briefly, leaves of 4-week-old *Arabidopsis *plants growing at 23°C with 12 h light were infiltrated with mock (10 mM MgCl_2_), non-pathogenic strain *Pst *DC3000 *hrcC*^-^, *Pst *DC3000 (empty vector) and *Pst *DC3000 (*avrRpt2*) at a concentration of 2 × 10^7 ^cfu/ml. The leaves were collected at 6 and 14 hours post-inoculation.

For abiotic stress treatment in *Arabidopsis*, plants grew at 23°C with 16 h light for 19 days, and were then divided into four groups. One of the groups was water-deprived (drought treatment) while the others were maintained with regular irrigation. At day 29, one group of plants was treated with salt solution (200 mM NaCl as irrigation solution for 24 h). Another group of plants was transferred to a growth chamber at 5°C and 16 h light for 24 h for cold treatment. The fourth group of plants was always maintained under regular conditions as a normal control. At day 30, all four groups of plants were collected. For small RNA library construction, the whole shoots of plants (stems, leaves and inflorescences) were used.

Three-month-old rice growing at 28°C and approximately 13 h of natural light were treated with drought (water withholding for 3 days), cold (5°C for 24 h) and salt (400 mM NaCl for 24 h, added as irrigation solution). The inflorescences of these plants were collected and used for sRNA library construction.

sRNA extraction and library construction were carried out as described previously [75,76]. Briefly, total RNA was isolated from infiltrated leaves and fractionated on 15% denaturing polyacrylamide gel. RNA molecules ranging from 18 to 26 nucleotides were excised and ligated to 5'- and 3'-RNA adaptors using T4 RNA ligase followed by RT-PCR and gel purification as described in the instructions from Illumina Inc. The small RNA libraries were sequenced by Illumina Inc. and UCR core facility.

### Northern blot analysis of siRNAs

We used LNA probes for small RNA Northern blot analysis to detect nat-siRNAs. A total of 100 μg total RNA was separated on a 17% acrylimide gel. The blots were probed at 39°C with PerfectHyb Plus Hybridization Buffer (Sigma-Aldrich, Saint Louis, MO, USA). The sequences of the oligos were listed in Additional file 7.

### Quantitative RT-PCR analysis of small RNA targets

For QRT-PCR analysis, 5 μg total DNaseI-treated (Invitrogen, Grand Island, NY, USA) RNA was used for synthesizing cDNA using Oligo dT and SuperScript II (Invitrogen). Amplification of small RNA targets was carried out using a real-time PCR machine (MyiQ, Bio-Rad, Hercules, CA, USA). The sequences of primers used here are listed in Additional file 7.

### Pre-processing of deep sequencing data

Raw sequence reads were parsed to remove 3'-adaptors using an in-house program. Unique sequences from *Arabidopsis *small-RNA libraries were mapped to the *Arabidopsis *chromosome sequences [77]. To produce candidate datasets of sRNAs, we removed sequencing reads that matched to rRNAs, tRNAs, snRNAs, snoRNAs, transposons, retrotransposons, or repeats. rRNA, snRNA and snoRNA sequences were obtained from TAIR8 genome annotation, and tRNA sequences were downloaded from the *Arabidopsis *tRNA database [78] and TAIR [77]. Transposons, retrotransposons, and repeat sequences were downloaded from the TIGR v2 *Arabidopsis *Repeat Database [79] and RepBase [80].

The rice genome and annotation were retrieved from the MSU Rice Genome Annotation Project V6.1 (RGAP 6.1). Non-coding RNA (tRNA, rRNA, snoRNA and snRNA) were collected from fRNAdb [81] and the UCSC tRNA database. Transposons, retrotransposons and repeat sequences were based on transposable elements of MSU RGAP 6.1 and RepBase [80].

### Analysis of enrichment of endogenous sRNAs in the *cis*-NATs

The enrichment of sRNAs in the overlapping regions of NATs was examined by the method outlined in [69]. The sRNAs in the combined libraries for *Arabidopsis *or rice were used here. Briefly, for an *l*-nucleotide genic or genomic region that gives rise to *n *sRNAs, we defined *n*/*l *as the density of small RNA loci in this region. For each pair of *cis*-NATs, we counted the number of sRNAs mapping to the overlapping region, *N_o_*, and the total number of sRNAs matching the non-overlapping regions of the two genes, *N_g_*, and measured the length of the overlapping region, *L*_o_, and the total length of non-overlapping regions of the two genes, *L_g_*. Then we computed the density of small-RNA loci in the overlapping region, *N_o_/L_o_*, and that of the non-overlapping regions of the two NAT genes, *N_g_/L*_g_. The one-tail paired two-sample *t*-test was applied to the two-paired samples of siRNA density, *N_o_/L_o _*and *N_g_/L*_g_. The average densities in the overlapping regions (*A_o_*) and in the non-overlapping regions of the NAT genes (*A_g_*) that spawn sRNAs were computed. The ratio *A_o_*/*A_g _*was considered as the enrichment score.

In order to explore whether *cis*-NATs were more likely to give rise to siRNAs than other protein-coding genes, we calculated the average densities in the overlapping regions (*A_o_*) of *cis*-NATs. We then calculated the average densities *A_u _*of a set of randomly chosen regions from the other protein-coding genes, which have the same size as the *cis*-NATs. Statistical significance was measured by a *P*-value computed as the frequency of the occurrences that *A_u _*was greater than *A_o_*. We applied this method to compare the siRNA density of convergent *cis*-NATs with that of 3'-UTRs and the siRNA density of divergent *cis*-NATs with that of 5'-UTRs.

To analyze the regulation of nat-siRNAs under a specific condition, the sRNA reads in a single library that matched 100% to the genome were normalized to one million. Then the normalized sRNA abundances were compared between different libraries. Furthermore, we calculated the maximum fold change among all treated samples compared to corresponding control conditions as a supplemental value (Additional files 10 and 11).

### Classification of nat-siRNAs

To classify the *cis*-NAT pairs into distributed and site-specific patterns, we first clustered sequencing reads mapped to regions of *cis*-NATs. Starting from the first reads closest to the 5' end of a *cis*-NAT, reads were clustered if the positions of their first nucleotides were within a ten-nucleotide long segment. Clusters with more than five reads were retained for further analysis. We calculated two metrics for each *cis*-NAT: 1) the number of small-RNA clusters and 2) the percentage of the number of reads within these clusters relative to the total number of reads mapped to the whole *cis*-NAT. We then categorized a *cis*-NAT to have a site-specific pattern if 1) it had no more than ten siRNA clusters and 2) the percentage of the reads within the clusters was greater than 50%; otherwise we categorized the *cis*-NAT to have a distributed pattern. The criterion of 10 siRNA clusters was chosen based on the observation that the frequency of clusters of 84 *Arabidopsis cis*-NATs has a clear separation between 10 and above (Additional file 5a). The percentage criterion (more than 50% sequence reads) was determined based on a visual exanimation of the relationship between the number of clusters and the frequencies of reads within the clusters in the 84 *cis*-NATS, as plotted Additional file 5b. As shown in the figure, this relationship seemed to follow two distributions, one is a linear correlation represented by the line in Additional file 5b and the other is a group with no more than ten clusters that retain more than 50% of the total sequence reads. The second distribution thus formed site-specific patterns of siRNAs. We thus used these two criteria because a site-specific pattern should have relatively more reads appearing in a relatively smaller number of clusters than a distributed pattern.

### Microarray data and data analysis

The processed microarray data of *dcl1*-*7 *and Ler WT *Arabidopsis *were downloaded from GEO accession number GSE2473 (samples GSM47014, GSM47015, GSM47016, GSM47017, GSM47018, GSM47019, GSM47023, GSM47024, GSM47025, GSM47026 and GSM47027). We identified differentially expressed genes using Rank Product [82]. The rationale behind Rank Product is that it is unlikely for a gene to be ranked in the top position in all replicates if the gene was not differentially expressed. It has been shown that Rank Product is less sensitive to noise and has a better performance than other methods when sample size is small [83]. We selected differentially expressed genes with a false discovery rate no greater than 0.05 for 1,000 permutations. Differentially expressed probe sets were then mapped to corresponding genes according to the annotation of the Affymatrix ATH1-121501 chip.

### Analysis of association of AGOs and nat-siRNAs

The processed AGO data for *Arabidopsis *were downloaded from the GEO databases under accession numbers GSE10036 and GSE12037. The number of AGO-associated nat-siRNAs was obtained by perfectly mapping the identified nat-siRNAs to each of the AGO pull-down datasets separately. The number was then normalized by the total number of AGO reads in an AGO dataset that can be perfectly mapped to the genome raised to per million for normalization. The enrichment of nat-siRNAs in an AGO pull-down relative to the other AGOs was calculated as the percentage of the normalized nat-siRNAs in the AGO of the total normalized nat-siRNAs in all AGO pull-downs.

### Identification of nat-siRNA regulated *cis*-NATs

The nat-siRNA regulated *cis*-NATs were identified from the set of differentially expressed genes from the microarray data of the *dcl1*-*7 *and Ler WT control *Arabidopsis *plants. The analysis was carried out based on two criteria: 1) more than 1.5-fold up-regulation with a *P*-value less than 0.05 in the *dcl1*-*7 *mutant and 2) presence of nat-siRNAs produced by the *cis*-NATs on the strands opposite to the NAT transcripts. The rationale behind these criteria is that the main function of DCL1-generated nat-siRNAs is to target *cis*-NATs on the opposite strands to down-regulate their expression. Thus, in a DCL1 mutant where no such nat-siRNAs are produced, the expressions of the targeted NATs are expected to be up-regulated.

## Abbreviations

AGO: Argonaute; *cis*-NAT: *cis*-natural antisense transcript; DCL: Dicer-like; dsRNA: double-stranded RNA; endo-siRNA: endogenous siRNA; GEO: Gene Expression Omnibus; hc-siRNA: heterochromatic siRNA; IR: inverted repeat; LNA: locked nucleic acid; lsiRNA: long siRNA; miRNA: microRNA; PCR: polymerase chain reaction; Pol: RNA polymerase; Pst: *Pseudomonas Syringae pv tomato*; RACE: rapid amplification of cDNA ends; RDR: RNA-dependent RNA polymerase; siRNA: small interfering RNA; snRNA: small nuclear RNA; snoRNA: small nucleolar RNA; sRNA: small RNA; ta-siRNA: trans-acting siRNA; UTR: untranslated region; WT: wild type.

## Authors' contributions

HJ initiated the project; HJ, WZ and JZ designed the experiments; XiaomingZ and BB carried out the experiments; JX, XuefengZ and CZ performed the computational analyses; SG, LL, DN, HZ, CL and TW participated in the experimental analyses. HJ, WZ, JZ, RL, YiL, WL and JG coordinated the research. HJ, XiaomingZ, YL, JX, JZ and WZ wrote the paper. All authors have read and approved the manuscript for publication.

## Supplementary Material

Additional file 1 **Distributions of the sequencing reads from small RNA libraries of abiotic and biotic treated *Arabidopsis *and abiotic challenged rice**. Shown in the table are the total number of raw sequencing reads (total), the number of qualified reads that can map perfectly to the corresponding *Arabidopsis *or rice genome (mapped), intergenic regions (intergenic), intronic sequences (introns), transposons or repeats (repeats/mobile), tRNA, rRNA, snoRNA and snRNA sequences (ncRNA), trans-acting siRNA (ta-siRNA), microRNA sequences (miRNAs), intron-exon junctions (intron-exon junction) and exon-exon junctions (exon-exon junction). No mismatches were allowed in the mapping. The second number for each condition (column) is the percentage of reads relative to the total mapped reads. Click here for file

Additional file 2 **(a-d) Distributions of the lengths (a, b) and the first nucleotides (c, d) of total siRNAs and nat-siRNAs in stress-challenged *Arabidopsis *and rice**. (a) Length distributions of unique sequencing reads in *Arabidopsis*. The blue and red bars represent total siRNAs and nat-siRNAs, respectively. (b) Length distributions of unique sequencing reads in rice. The blue and red bars represent total siRNAs and nat-siRNAs, respectively. (c) First-nucleotide distribution of unique sequencing reads in *Arabidopsis*. (d) First-nucleotide distribution of unique sequencing reads in rice. Click here for file

Additional file 3 **Normalized reads of siRNAs mapped to the overlapping regions of *Arabidopsis *and rice *cis*-NAT pairs, respectively (a) siRNAs mapped to the overlapping regions of 84 *Arabidopsis cis*-NAT pairs**. **(b) **siRNAs mapped to the overlapping regions of 119 rice *cis*-NAT pairs. Each small RNA library was normalized to one million reads with 100% matching to the genome. The sum of normalized reads from each library was listed for each *cis*-NAT pair. Click here for file

Additional file 4 ***Arabidopsis cis*-NATs with two classes of small RNAs mapped to the overlapping regions**. 'gene1' and 'gene2' represent the first and second transcript in each *cis*-NAT pair. Listed are 84 *Arabidopsis cis*-NATs with siRNAs mapped to the overlapping region. Raw reads of 20- to 22-nucleotide and 23- to 28-nucleotide classes of siRNAs matching 100% to the genome were analyzed. The percentage of 20- to 22-nucleotide and 23- to 28-nucleotide siRNAs are presented. Click here for file

Additional file 5 **(a) Distributions of the number of small RNA clusters in 84 *Arabidopsis cis*-NATs**. The red line represents the separation between site-specific (left) and distributed (right) patterns. **(b) **The plot of two metrics of 84 *cis*-NATs in *Arabidopsis*. Each dot represents the number of clusters within the *cis*-NAT whole region and the percentage of small RNA reads in all clusters. The red dots in the rectangle were classified as site-specific patterns, whereas the blue dots were distributed patterns, represented by a linear correlated line. Click here for file

Additional file 6 **Classification of *cis*-NAT pairs**. We analyzed 84 *Arabidopsis *and 119 rice *cis*-NAT pairs that have more than 10 raw siRNAs mapped to the overlap region. The reads analyzed here are the raw sequencing reads mapped to the whole region from the combination of all libraries. **(a) ***Arabidopsis cis*-NAT pairs display different distribution patterns. **(b) **Rice *cis*-NAT pairs display different distribution patterns. Click here for file

Additional file 7 **Oligos used in this study**. A plus sign ('*+*') before the nucleotide depicts LNA residues. Click here for file

Additional file 8 **Small RNAs mapped to the introns or intron-exon junction regions of *Arabidopsis cis*-NATs**. Copy number indicates the number of raw reads in the combination of all libraries. **(a) **siRNAs mapping to introns in the overlapping region of *cis*-NATs in *Arabidopsis*. **(b) **siRNAs mapping to introns in the whole region of *cis*-NATs in *Arabidopsis*. Click here for file

Additional file 9 **Small RNAs mapped to the introns or intron-exon junction regions of rice *cis*-NATs**. Raw reads from all the libraries were analyzed. **(a) **siRNAs mapping to introns in the overlapping region of *cis*-NATs in rice. **(b) **siRNAs mapping to introns in the whole region of *cis*-NATs in rice Click here for file

Additional file 10 **Normalized reads of siRNAs mapped to 84 *Arabidopsis *NATs under different conditions**. Each library was normalized to a million reads that perfectly mapped to the genome. The fold change with maximum absolute value between treated sample and corresponding control is listed as a supplemental value. The pairs with more than two-fold change were annotated in red (up) or in blue (down). **(a) **Reads of small RNAs mapped to overlapping region of NATs under different conditions. **(b) **Reads of small RNAs mapped to whole region of NAT under different conditions. Click here for file

Additional file 11 **Normalized reads of siRNAs mapped to 119 rice NATs under different conditions**. siRNAs matching 100% to the genome were analyzed and each library was normalized to a million reads before analyzing. The fold change with maximum absolute value between treated and untreated libraries is listed. The pairs with more than a two-fold change are displayed in red (up, +) or blue (down, -). **(a) **Reads of small RNAs mapped to overlapping region of NATs under different conditions. **(b) **Reads of small RNAs mapped to whole region of NATs under different conditions. Click here for file

Additional file 12 ***Arabidopsis cis*-NATs that are up-regulated in the *dcl1*-*7 *mutant**. **(a) **List of probes of the 93 genes of the 84 *cis*-NAT pairs. Annotations of the Affymetrix ATH1-121501 chip were used and no probes were found for the rest of the *cis*-NAT genes. **(b) **The 23 *cis*-NAT genes up-regulated in *dcl1*-*7*. Click here for file

Additional file 13 **Expression analysis of NAT transcripts in the *fwf2 *single mutant**. The expression of NAT transcripts was analyzed by quantitative RT-PCR. Total RNA (5 μg) was used for DNase treatment and reverse transcription. Error bars indicate the technical replicates and similar results were obtained from two biological repeats. Click here for file

Additional file 14 **Association of 20- to 22-nucleotide and 23- to 26-nucleotide classes of nat-siRNAs with different AGOs in *Arabidopsis***. Unique reads of nat-siRNAs associated with AGO1, AGO2, AGO4 and AGO7 were analyzed. Loaded reads represent unique reads of nat-siRNAs that were loaded into distinct AGOs. The percentage in total nat-siRNA-AGO libraries indicates the distribution of loaded nat-siRNAs in different AGOs. Click here for file

Additional file 15 **SRO5-P5CDH siRNAs derived from salt and cold stress challenged *Arabidopsis***. The siRNAs were identified from GEO database accession number GSE33642. **(a) **Reads of siRNAs positively/negatively match to At5G62520 (SRO5) in the overlap/non-overlap region. The '+ strand' and '- strand' indicate positively or negatively matching. **(b) **Distribution pattern of SRO5-P5DCH siRNAs. siRNAs positively or negatively matched to At5G62520 are displayed above or below the SRO5-P5DCH gene pair. Exons and introns are represented by red and blue dishes, respectively. The overlapping region is indicated by the two green lines. Click here for file

## References

[B1] Baulcombe D RNA silencing in plants. Nature 2004 431 356 363 10.1038/nature02874 15372043

[B2] Matzke MA Birchler JA RNAi-mediated pathways in the nucleus. Nat Rev Genet 2005 6 24 35 10.1038/nrg1500 15630419

[B3] Chapman EJ Carrington JC Specialization and evolution of endogenous small RNA pathways. Nat Rev Genet 2007 8 884 896 10.1038/nrg2179 17943195

[B4] Zamore PD Haley B Ribo-gnome: The big world of small RNAs. Science 2005 309 1519 1524 10.1126/science.1111444 16141061

[B5] Jones-Rhoades MW Bartel DP Bartel B MicroRNAs and their regulatory roles in plants. Annu Rev Plant Biol 2006 57 19 53 10.1146/annurev.arplant.57.032905.105218 16669754

[B6] Mosher RA Melnyk CW Kelly KA Dunn RM Studholme DJ Baulcombe DC Uniparental expression of PolIV-dependent siRNAs in developing endosperm of *Arabidopsis*. Nature 2009 460 283 U151 10.1038/nature08084 19494814

[B7] Pikaard CS Haag JR Ream T Wierzbicki AT Roles of RNA polymerase IV in gene silencing. Trends Plant Sci 2008 13 390 397 10.1016/j.tplants.2008.04.008 18514566 PMC2679257

[B8] Wierzbicki AT Haag JR Pikaard CS Noncoding transcription by RNA polymerase Pol IVb/Pol V mediates transcriptional silencing of overlapping and adjacent genes. Cell 2008 135 635 648 10.1016/j.cell.2008.09.035 19013275 PMC2602798

[B9] Vazquez F *Arabidopsis *endogenous small RNAs: highways and byways. Trends Plant Sci 2006 11 460 468 10.1016/j.tplants.2006.07.006 16893673

[B10] Matzke M Kanno T Daxinger L Huettel B Matzke AJ RNA-mediated chromatin-based silencing in plants. Curr Opin Cell Biol 2009 21 367 376 10.1016/j.ceb.2009.01.025 19243928

[B11] Wierzbicki AT Ream TS Haag JR Pikaard CS RNA polymerase v transcription guides ARGONAUTE4 to chromatin. Nat Genet 2009 41 630 634 10.1038/ng.365 19377477 PMC2674513

[B12] Zaratiegui M Irvine DV Martienssen RA Noncoding RNAs and gene silencing. Cell 2007 128 763 776 10.1016/j.cell.2007.02.016 17320512

[B13] Katiyar-Agarwal S Gao S Vivian-Smith A Jin H A novel class of bacteria-induced small RNAs in *Arabidopsis*. Genes Dev 2007 21 3123 3134 10.1101/gad.1595107 18003861 PMC2081978

[B14] Ron M Saez MA Williams LE Fletcher JC McCormick S Proper regulation of a sperm-specific *cis*-nat-siRNA is essential for double fertilization in *Arabidopsis*. Genes Dev 2010 24 1010 1021 10.1101/gad.1882810 20478994 PMC2867206

[B15] Zubko E Meyer P A natural antisense transcript of the Petunia hybrida Sho gene suggests a role for an antisense mechanism in cytokinin regulation. Plant J 2007 52 1131 1139 10.1111/j.1365-313X.2007.03309.x 17944812 PMC2253869

[B16] Katiyar-Agarwal S Morgan R Dahlbeck D Borsani O Villegas A Zhu JK Staskawicz BJ Jin HL A pathogen-inducible endogenous siRNA in plant immunity. Proc Natl Acad Sci USA 2006 103 18002 18007 10.1073/pnas.0608258103 17071740 PMC1693862

[B17] Borsani O Zhu JH Verslues PE Sunkar R Zhu JK Endogenous siRNAs derived from a pair of natural *cis*-antisense transcripts regulate salt tolerance in *Arabidopsis*. Cell 2005 123 1279 1291 10.1016/j.cell.2005.11.035 16377568 PMC3137516

[B18] Jin H Endogenous small RNAs and antibacterial immunity in plants. FEBS Lett 2008 582 2679 2684 10.1016/j.febslet.2008.06.053 18619960 PMC5912937

[B19] Jin H Vacic V Girke T Lonardi S Zhu JK Small RNAs and the regulation of *cis*-natural antisense transcripts in *Arabidopsis*. BMC Mol Biol 2008 9 6 10.1186/1471-2199-9-6 18194570 PMC2262095

[B20] Czech B Malone CD Zhou R Stark A Schlingeheyde C Dus M Perrimon N Kellis M Wohlschlegel JA Sachidanandam R Hannon GJ Brennecke J An endogenous small interfering RNA pathway in *Drosophila*. Nature 2008 453 798 U797 10.1038/nature07007 18463631 PMC2895258

[B21] Watanabe T Totoki Y Toyoda A Kaneda M Kuramochi-Miyagawa S Obata Y Chiba H Kohara Y Kono T Nakano T Surani MA Sakaki Y Sasaki H Endogenous siRNAs from naturally formed dsRNAs regulate transcripts in mouse oocytes. Nature 2008 453 539 U539 10.1038/nature06908 18404146

[B22] Ghildiyal M Seitz H Horwich MD Li CJ Du TT Lee S Xu J Kittler ELW Zapp ML Weng ZP Zamore PD Endogenous siRNAs derived from transposons and mRNAs in *Drosophila *somatic cells. Science 2008 320 1077 1081 10.1126/science.1157396 18403677 PMC2953241

[B23] Okamura K Balla S Martin R Liu N Lai EC Two distinct mechanisms generate endogenous siRNAs from bidirectional transcription in *Drosophila *melanogaster. Nat Struct Mol Biol 2008 15 581 590 10.1038/nsmb.1438 18500351 PMC2713754

[B24] Okamura K Robine N Liu Y Liu Q Lai EC R2D2 organizes small regulatory RNA pathways in *Drosophila*. Mol Cell Biol 2011 31 884 896 10.1128/MCB.01141-10 21135122 PMC3028645

[B25] Drinnenberg IA Weinberg DE Xie KT Mower JP Wolfe KH Fink GR Bartel DP RNAi in budding yeast. Science 2009 326 544 550 10.1126/science.1176945 19745116 PMC3786161

[B26] Cai P Hou N Piao X Liu S Liu H Yang F Wang J Jin Q Wang H Chen Q Profiles of small non-coding RNAs in *Schistosoma japonicum *during development. PLoS Negl Trop Dis 2011 5 e1256 10.1371/journal.pntd.0001256 21829742 PMC3149011

[B27] Henz SR Cumbie JS Kasschau KD Lohmann JU Carrington JC Weigel D Schmid M Distinct expression patterns of natural antisense transcripts in arabidopsis. Plant Physiol 2007 144 1247 1255 10.1104/pp.107.100396 17496106 PMC1914114

[B28] Chellappan P Xia J Zhou X Gao S Zhang X Coutino G Vazquez F Zhang W Jin H siRNAs from miRNA sites mediate DNA methylation of target genes. Nucleic Acids Res 2010 38 6883 6894 10.1093/nar/gkq590 20621980 PMC2978365

[B29] Zhang W Gao S Zhou X Xia J Chellappan P Zhou X Zhang X Jin H Multiple distinct small RNAs originated from the same microRNA precursors. Genome Biol 2010 11 R81 10.1186/gb-2010-11-8-r81 20696037 PMC2945783

[B30] Zhang W Gao S Zhou X Chellappan P Chen Z Zhou X Zhang X Fromuth N Coutino G Coffey M Jin H Bacteria-responsive microRNAs regulate plant innate immunity by modulating plant hormone networks. Plant Mol Biol 2011 75 93 105 10.1007/s11103-010-9710-8 21153682 PMC3005105

[B31] Chen H-M Chen L-T Patel K Li Y-H Baulcombe DC Wu S-H 22-nucleotide RNAs trigger secondary siRNA biogenesis in plants. Proc Natl Acad Sci USA 2010 107 15269 15274 10.1073/pnas.1001738107 20643946 PMC2930544

[B32] Cuperus JT Carbonell A Fahlgren N Garcia-Ruiz H Burke RT Takeda A Sullivan CM Gilbert SD Montgomery TA Carrington JC Unique functionality of 22-nt miRNAs in triggering RDR6-dependent siRNA biogenesis from target transcripts in *Arabidopsis*. Nat Struct Mol Biol 2010 17 997 U111 10.1038/nsmb.1866 20562854 PMC2916640

[B33] Wang H Zhang XR Liu J Kiba T Woo J Ojo T Hafner M Tuschl T Chua NH Wang XJ Deep sequencing of small RNAs specifically associated with *Arabidopsis *AGO1 and AGO4 uncovers new AGO functions. Plant J 2011 67 292 304 10.1111/j.1365-313X.2011.04594.x 21457371 PMC3135789

[B34] Tam OH Aravin AA Stein P Girard A Murchison EP Cheloufi S Hodges E Anger M Sachidanandam R Schultz RM Hannon GJ Pseudogene-derived small interfering RNAs regulate gene expression in mouse oocytes. Nature 2008 453 534 U538 10.1038/nature06904 18404147 PMC2981145

[B35] Hofacker IL Vienna RNA secondary structure server. Nucleic Acids Res 2003 31 3429 3431 10.1093/nar/gkg599 12824340 PMC169005

[B36] Lu C Jeong DH Kulkarni K Pillay M Nobuta K German R Thatcher SR Maher C Zhang L Ware D Liu B Cao X Meyers BC Green PJ Genome-wide analysis for discovery of rice microRNAs reveals natural antisense microRNAs (nat-miRNAs). Proc Natl Acad Sci USA 2008 105 4951 4956 10.1073/pnas.0708743105 18353984 PMC2290808

[B37] Kasschau KD Fahlgren N Chapman EJ Sullivan CM Cumbie JS Givan SA Carrington JC Genome-wide profiling and analysis of *Arabidopsis *siRNAs. PLoS Biol 2007 5 e57 10.1371/journal.pbio.0050057 17298187 PMC1820830

[B38] Laubinger S Zeller G Henz SR Buechel S Sachsenberg T Wang J-W Raetsch G Weigel D Global effects of the small RNA biogenesis machinery on the *Arabidopsis thaliana *transcriptome. Proc Natl Acad Sci USA 2010 107 17466 17473 10.1073/pnas.1012891107 20870966 PMC2955092

[B39] Goetz M Vivian-Smith A Johnson SD Koltunow AM AUXIN RESPONSE FACTOR8 is a negative regulator of fruit initiation in *Arabidopsis*. Plant Cell 2006 18 1873 1886 10.1105/tpc.105.037192 16829592 PMC1533983

[B40] Goetz M Hooper LC Johnson SD Rodrigues JCM Vivian-Smith A Koltunow AM Expression of aberrant forms of AUXIN RESPONSE FACTOR8 stimulates parthenocarpy in *Arabidopsis *and tomato. Plant Physiol 2007 145 351 366 10.1104/pp.107.104174 17766399 PMC2048734

[B41] Katiyar-Agarwal S Gao S Vivian-Smith A Jin H A novel class of bacteria-induced small RNAs in *Arabidopsis*. Genes Dev 2007 21 3123 3134 10.1101/gad.1595107 18003861 PMC2081978

[B42] Jay F Wang Y Yu A Taconnat L Pelletier S Colot V Renou J-P Voinnet O Misregulation of AUXIN RESPONSE FACTOR 8 underlies the developmental abnormalities caused by three distinct viral silencing suppressors in *Arabidopsis*. PLoS Pathogens 2011 7 e1002035 10.1371/journal.ppat.1002035 21589905 PMC3093370

[B43] Mi SJ Cai T Hu YG Chen Y Hodges E Ni FR Wu L Li S Zhou H Long CZ Chen S Hannon GJ Qi YJ Sorting of small RNAs into *Arabidopsis *argonaute complexes is directed by the 5 ' terminal nucleotide. Cell 2008 133 116 127 10.1016/j.cell.2008.02.034 18342361 PMC2981139

[B44] Montgomery TA Howell MD Cuperus JT Li D Hansen JE Alexander AL Chapman EJ Fahlgren N Allen E Carrington JC Specificity of ARGONAUTE7-miR390 interaction and dual functionality in TAS3 trans-acting siRNA formation. Cell 2008 133 128 141 10.1016/j.cell.2008.02.033 18342362

[B45] Jaubert M Bhattacharjee S Mello AFS Perry KL Moffett P ARGONAUTE2 Mediates RNA-Silencing Antiviral Defenses against Potato virus X in *Arabidopsis*. Plant Physiol 2011 156 1556 1564 10.1104/pp.111.178012 21576511 PMC3135937

[B46] Zhang XM Zhao HW Gao S Wang WC Katiyar-Agarwal S Huang HD Raikhel N Jin HL *Arabidopsis *Argonaute 2 regulates innate immunity via miRNA393*-mediated silencing of a golgi-localized SNARE gene, MEMB12. Mol Cell 2011 42 356 366 10.1016/j.molcel.2011.04.010 21549312 PMC3101262

[B47] Harvey JJW Lewsey MG Patel K Westwood J Heimstadt S Carr JP Baulcombe DC An antiviral defense role of AGO2 in plants. PLoS One 2011 6 e14639 10.1371/journal.pone.0014639 21305057 PMC3031535

[B48] Wang XB Jovel J Udomporn P Wang Y Wu QF Li WX Gasciolli V Vaucheret H Ding SW The 21-nucleotide, but not 22-nucleotide, viral secondary small interfering RNAs direct potent antiviral defense by two cooperative Argonautes in *Arabidopsis thaliana*. Plant Cell 2011 23 1625 1638 10.1105/tpc.110.082305 21467580 PMC3101545

[B49] Vaucheret H Plant ARGONAUTES. Trends Plant Sci 2008 13 350 358 10.1016/j.tplants.2008.04.007 18508405

[B50] Zilberman D Cao X Jacobsen SE ARGONAUTE4 control of locus-specific siRNA accumulation and DNA and histone methylation. Science 2003 299 716 719 10.1126/science.1079695 12522258

[B51] Hamilton AJ Baulcombe DC A species of small antisense RNA in posttranscriptional gene silencing in plants. Science 1999 286 950 952 10.1126/science.286.5441.950 10542148

[B52] Llave C Kasschau KD Rector MA Carrington JC Endogenous and silencing-associated small RNAs in plants. Plant Cell 2002 14 1605 1619 10.1105/tpc.003210 12119378 PMC150710

[B53] Dunoyer P Brosnan CA Schott G Wang Y Jay F Alioua A Himber C Voinnet O An endogenous, systemic RNAi pathway in plants. EMBO J 2010 29 1699 1712 10.1038/emboj.2010.65 20414198 PMC2876969

[B54] Hamilton A Voinnet O Chappell L Baulcombe D Two classes of short interfering RNA in RNA silencing. EMBO J 2002 21 4671 4679 10.1093/emboj/cdf464 12198169 PMC125409

[B55] Dunoyer P Lecellier CH Parizotto EA Himber C Voinnet O Probing the microRNA and small interfering RNA pathways with virus-encoded suppressors of RNA silencing. Plant Cell 2004 16 1235 1250 10.1105/tpc.020719 15084715 PMC423212

[B56] Mlotshwa S Pruss GJ Peragine A Endres MW Li J Chen X Poethig RS Bowman LH Vance V DICER-LIKE2 plays a primary role in transitive silencing of transgenes in *Arabidopsis*. Plos One 2008 3 e1755 10.1371/journal.pone.0001755 18335032 PMC2262140

[B57] Fusaro AF Matthew L Smith NA Curtin SJ Dedic-Hagan J Ellacott GA Watson JM Wang M-B Brosnan C Carroll BJ Waterhouse PM RNA interference-inducing hairpin RNAs in plants act through the viral defence pathway. EMBO Rep 2006 7 1168 1175 10.1038/sj.embor.7400837 17039251 PMC1679793

[B58] Dunoyer P Himber C Voinnet O DICER-LIKE 4 is required for RNA interference and produces the 21-nucleotide small interfering RNA component of the plant cell-to-cell silencing signal. Nat Genet 2005 37 1356 1360 10.1038/ng1675 16273107

[B59] Dunoyer P Himber C Ruiz-Ferrer V Alioua A Voinnet O Intra- and intercellular RNA interference in *Arabidopsis thaliana *requires components of the microRNA and heterochromatic silencing pathways. Nat Genet 2007 39 848 856 10.1038/ng2081 17558406

[B60] Bouche N Lauressergues D Gasciolli V Vaucheret H An antagonistic function for *Arabidopsis *DCL2 in development and a new function for DCL4 in generating viral siRNAs. EMBO J 2006 25 3347 3356 10.1038/sj.emboj.7601217 16810317 PMC1523179

[B61] Ding SW Voinnet O Antiviral immunity directed by small RNAs. Cell 2007 130 413 426 10.1016/j.cell.2007.07.039 17693253 PMC2703654

[B62] Deleris A Gallego-Bartolome J Bao J Kasschau KD Carrington JC Voinnet O Hierarchical action and inhibition of plant Dicer-like proteins in antiviral defense. Science 2006 313 68 71 10.1126/science.1128214 16741077

[B63] Diaz-Pendon JA Li F Li W-X Ding S-W Suppression of antiviral silencing by cucumber mosaic virus 2b protein in *Arabidopsis *is associated with drastically reduced accumulation of three classes of viral small interfering RNAs. Plant Cell 2007 19 2053 2063 10.1105/tpc.106.047449 17586651 PMC1955711

[B64] Garcia-Ruiz H Takeda A Chapman EJ Sullivan CM Fahlgren N Brempelis KJ Carrington JC *Arabidopsis *RNA-dependent RNA polymerases and Dicer-like proteins in antiviral defense and small interfering RNA biogenesis during turnip mosaic virus infection. Plant Cell 2010 22 481 496 10.1105/tpc.109.073056 20190077 PMC2845422

[B65] Wu L Zhou H Zhang Q Zhang J Ni F Liu C Qi Y DNA methylation mediated by a MicroRNA pathway. Mol Cell 2010 38 465 475 10.1016/j.molcel.2010.03.008 20381393

[B66] Zheng X Zhu J Kapoor A Zhu J-K Role of *Arabidopsis *AGO6 in siRNA accumulation, DNA methylation and transcriptional gene silencing. EMBO J 2007 26 1691 1701 10.1038/sj.emboj.7601603 17332757 PMC1829372

[B67] Carlile M Swan D Jackson K Preston-Fayers K Ballester B Flicek P Werner A Strand selective generation of endo-siRNAs from the Na/phosphate transporter gene Slc34a1 in murine tissues. Nucleic Acids Res 2009 37 2274 2282 10.1093/nar/gkp088 19237395 PMC2673434

[B68] Carlile M Nalbant P Preston-Fayers K McHaffie GS Werner A Processing of naturally occurring sense/antisense transcripts of the vertebrate Slc34a gene into short RNAs. Physiol Genomics 2008 34 95 100 10.1152/physiolgenomics.00004.2008 18413783

[B69] Zhou X Sunkar R Jin H Zhu J-K Zhang W Genome-wide identification and analysis of small RNAs originated from natural antisense transcripts in Oryza sativa. Genome Res 2009 19 70 78 18971307 10.1101/gr.084806.108 PMC2612963

[B70] Wang XJ Gaasterland T Chua NH Genome-wide prediction and identification of *cis*-natural antisense transcripts in *Arabidopsis thaliana*. Genome Biol 2005 6 R30 10.1186/gb-2005-6-4-r30 15833117 PMC1088958

[B71] Werner A Berdal A Natural antisense transcripts: sound or silence? Physiol Genomics 2005 23 125 131 10.1152/physiolgenomics.00124.2005 16230481

[B72] Qi YJ He XY Wang XJ Kohany O Jurka J Hannon GJ Distinct catalytic and non-catalytic roles of ARGONAUTE4 in RNA-directed DNA methylation. Nature 2006 443 1008 1012 10.1038/nature05198 16998468

[B73] He Y Vogelstein B Velculescu VE Papadopoulos N Kinzler KW The antisense transcriptomes of human cells. Science 2008 322 1855 1857 10.1126/science.1163853 19056939 PMC2824178

[B74] Smalheiser NR Lugli G Thimmapuram J Cook EH Larson J Endogenous siRNAs and noncoding RNA-derived small RNAs are expressed in adult mouse hippocampus and are up-regulated in olfactory discrimination training. RNA 2011 17 166 181 10.1261/rna.2123811 21045079 PMC3004058

[B75] Katiyar-Agarwal S Jin H Discovery of pathogen-regulated small RNAs in plants. Methods Enzymol 2007 427 215 227 17720487 10.1016/S0076-6879(07)27012-0

[B76] Chellappan P Jin H Discovery of plant microRNAs and short-interfering RNAs by deep parallel sequencing. Methods Mol Biol 2009 495 121 132 10.1007/978-1-59745-477-3_11 19085152

[B77] TAIR8 genome annotation http://www.Arabidopsis.org

[B78] *Arabidopsis *tRNA Database http://lowelab.ucsc.edu/GtRNAdb/Athal

[B79] TIGR v2 *Arabidopsis *Repeat Database ftp://ftp.plantbiology.msu.edu/pub/data/TIGR_Plant_Repeats

[B80] Repbase http://www.girinst.org/repbase/index.html

[B81] fRNAdb http://www.ncrna.org/frnadb/

[B82] Breitling R Armengaud P Amtmann A Herzyk P Rank products: a simple, yet powerful, new method to detect differentially regulated genes in replicated microarray experiments. Febs Lett 2004 573 83 92 10.1016/j.febslet.2004.07.055 15327980

[B83] Jeffery IB Higgins DG Culhane AC Comparison and evaluation of methods for generating differentially expressed gene lists from microarray data. BMC Bioinformatics 2006 7 359 10.1186/1471-2105-7-359 16872483 PMC1544358

