# Supplementary figures and images for: Genome-wide analysis of plant nat-siRNAs reveals insights into their distribution, biogenesis and function
Source: Genome Biol. 2012 Mar 22;13(3):R20. doi: 10.1186/gb-2012-13-3-r20 (PMC3439971; doi:10.1186/gb-2012-13-3-r20)

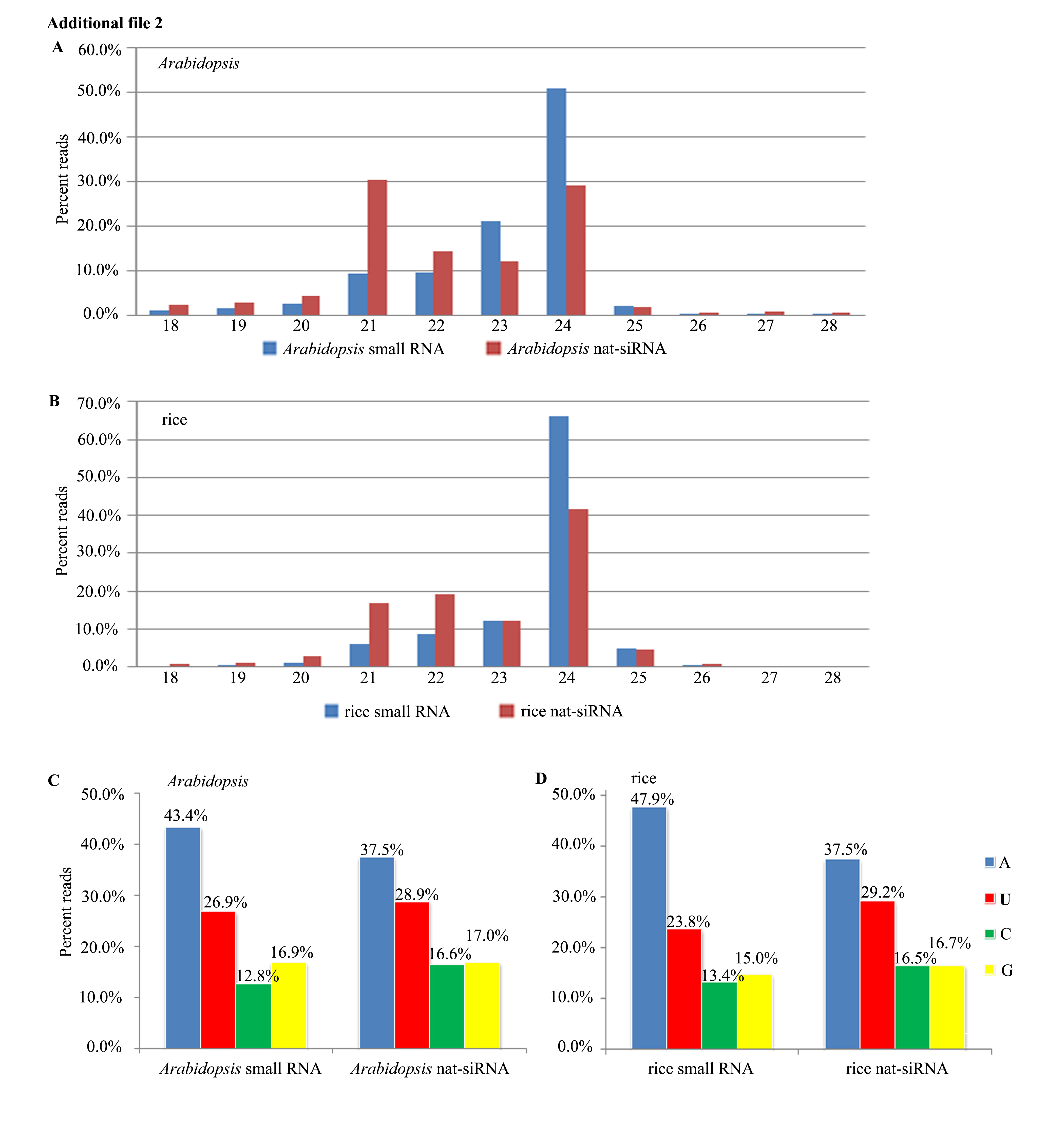

Supplement: Additional file 2 — (a-d) Distributions of the lengths (a, b) and the first nucleotides (c, d) of total siRNAs and nat-siRNAs in stress-challenged Arabidopsis and rice. (a) Length distributions of unique sequencing reads in Arabidopsis. The blue and red bars represent total siRNAs and nat-siRNAs, respectively. (b) Length distributions of unique sequencing reads in rice. The blue and red bars represent total siRNAs and nat-siRNAs, respectively. (c) First-nucleotide distribution of unique sequencing reads in Arabidopsis. (d) First-nucleotide distribution of unique sequencing reads in rice. [file gb-2012-13-3-r20-S2.JPEG]

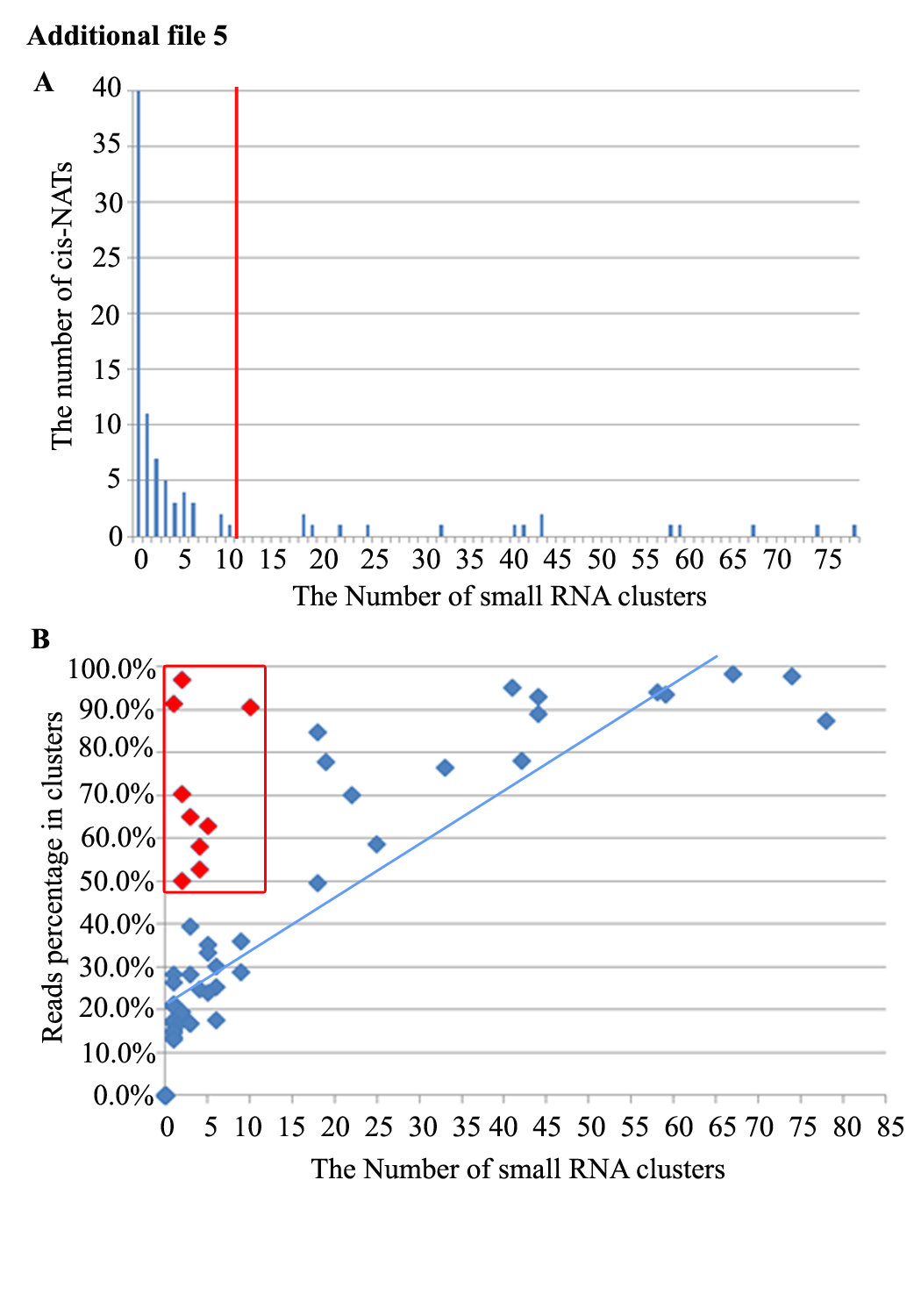

Supplement: Additional file 5 — (a) Distributions of the number of small RNA clusters in 84 Arabidopsis cis-NATs. The red line represents the separation between site-specific (left) and distributed (right) patterns. (b) The plot of two metrics of 84 cis-NATs in Arabidopsis. Each dot represents the number of clusters within the cis-NAT whole region and the percentage of small RNA reads in all clusters. The red dots in the rectangle were classified as site-specific patterns, whereas the blue dots were distributed patterns, represented by a linear correlated line. [file gb-2012-13-3-r20-S5.JPEG]

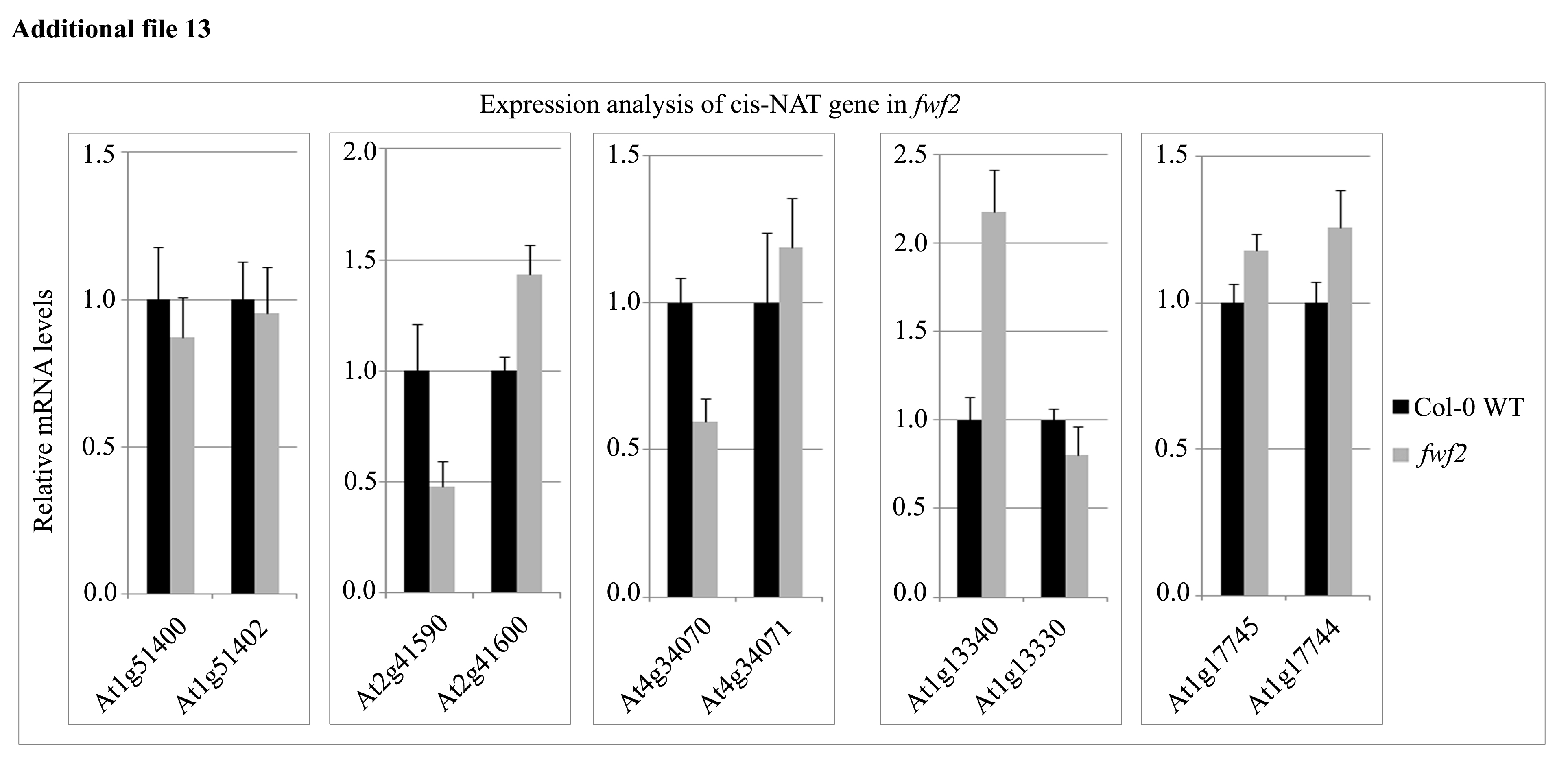

Supplement: Additional file 13 — Expression analysis of NAT transcripts in the fwf2 single mutant. The expression of NAT transcripts was analyzed by quantitative RT-PCR. Total RNA (5 μg) was used for DNase treatment and reverse transcription. Error bars indicate the technical replicates and similar results were obtained from two biological repeats. [file gb-2012-13-3-r20-S13.JPEG]

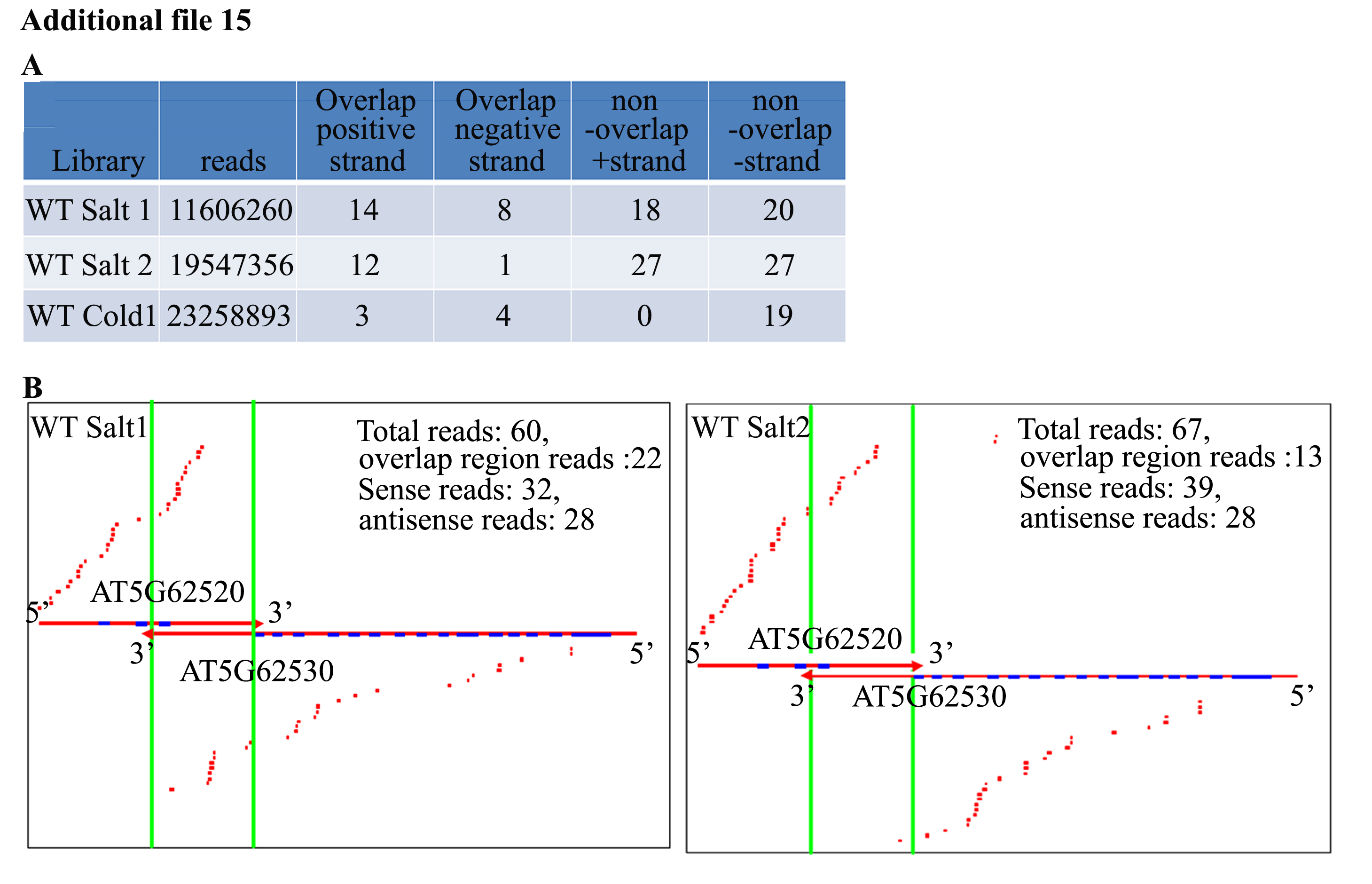

Supplement: Additional file 15 — SRO5-P5CDH siRNAs derived from salt and cold stress challenged Arabidopsis. The siRNAs were identified from GEO database accession number GSE33642. (a) Reads of siRNAs positively/negatively match to At5G62520 (SRO5) in the overlap/non-overlap region. The '+ strand' and '- strand' indicate positively or negatively matching. (b) Distribution pattern of SRO5-P5DCH siRNAs. siRNAs positively or negatively matched to At5G62520 are displayed above or below the SRO5-P5DCH gene pair. Exons and introns are represented by red and blue dishes, respectively. The overlapping region is indicated by the two green lines. [file gb-2012-13-3-r20-S15.JPEG]
